# Does Proximal Humerus Inter Locking System (PHILOS) Plating Provide a Good Functional Outcome in Proximal Humerus Fractures?

**DOI:** 10.7759/cureus.26474

**Published:** 2022-06-30

**Authors:** Prabhu Ethiraj, Sagar Venkataraman, Karthik S J, Arun H Shanthappa, Sandesh Agarawal

**Affiliations:** 1 Orthopaedics, Sri Devaraj Urs Medical College, Sri Devaraj Urs Academy of Higher Education and Research, Kolar, IND

**Keywords:** neer's classification, radiological union, constant score, philos, proximal humerus fracture

## Abstract

Introduction

There is still a lot of controversy and ambiguity, and no single therapy regimen or strategy for proximal humerus fractures has been proved to be uniformly helpful. According to estimates, only about 20% of proximal humeral fractures require surgery. This study aims at evaluating the functional outcome of treatment of Proximal humerus fractures with PHILOS (Proximal Humerus Inter Locking System) plating using Constant score and assessing the duration for fracture union by follow-up x-rays.

Methodology

This is a retrospective study on patients admitted to the Department of Orthopaedics attached to a tertiary hospital in Kolar from January 2017 to January 2020. After meeting inclusion and exclusion criteria, 40 patients were included in this study. All the patients underwent PHILOS plating after the pre-anesthetic check-up. The patients were regularly followed up, the functional outcome was assessed using the Constant Murley score, and the fracture union was assessed radiologically by follow-up x-rays.

Results

The mean age of our forty patients was 46.8 (20-70), with a male: female ratio of 2.3:1. Fractures united at an average of 13.75 weeks. The functional outcome was found to be excellent in 2 patients (5%), good in 22 patients (55%), fair in 7 patients (17.5%), and poor in 9 patients (22.5%). The Constant mean score achieved was 68.75±14.03. The mean time of fracture union was 13.75 weeks. The constant score was compared between variables like age groups, sex, fracture pattern, and mode of injury, but the values were statistically insignificant.

Conclusion

Even in individuals with osteopenic bones, we achieved excellent to good fracture repair in most cases. The locking plate provides adequate fracture stability and facilitates early shoulder mobilization without hindering fracture union.

## Introduction

There is still a lot of controversy and ambiguity, and no single therapy regimen or strategy for proximal humerus fractures has been proved to be uniformly helpful. Surgical treatment is rarely necessary because proximal humeral fractures are rarely displaced or angulated. According to estimates, only about 20% of proximal humeral fractures require surgery [1]. The need to treat such a fracture is determined by the fracture pattern, bone quality, and the patient's age and activity level [2]. The aim is to achieve stability and near-anatomical reduction as soon as conceivable so that mobilization can commence. The humeral head, greater tuberosity, lesser tuberosity, and proximal shaft are all involved in the proximal humerus fractures. Proximal humerus fractures account for about 4% of all fractures and 26% of all humerus fractures, and it usually affects people older than 65 years.

Due to the dislocation position and the patients' age, these fractures continue to complicate the surgeon. It can be treated with either surgical or conservative approaches, and the degree of displacement and angulation of fracture fragments determine which of the two procedures is used. Furthermore, because age is a major factor influencing management decisions, several complications arise in the functional approach. Osteoporotic bone, angular instability, implant impingement, bone loss, loss of reduction, and screw backing are frequent difficulties seen after the operative procedure of proximal humerus fractures [3,4].

20% of proximal humerus fractures necessitate surgery, and the Proximal Humerus Internal Locking System (PHILOS) has become the most often utilized treatment method. The incidence of proximal humerus fracture is more significant among the younger generation than the elderly cases [5]. This study aims to evaluate the functional outcome of Proximal humerus fractures treated with PHILOS plating using a Constant score and assess the fracture union duration by follow-up x-rays.

## Materials and methods

This is a retrospective study on patients admitted to the Department of Orthopaedics attached to a tertiary hospital in Kolar from January 2017 to January 2020. The Institutional Ethical Committee of Sri Devaraj Urs medical college approved the study with approval no- DMC/KLR/IEC/366/2021-22.

Patients more than 18 years of age who presented with acute displaced proximal humerus fractures and underwent a Proximal Humerus Interlocking System were included in the study. Pathological fracture, an association of fracture of ipsilateral scapula or clavicle, an association of pre-injury shoulder pathology, and an association of neurovascular injury were excluded. A total of 40 patients were included in this study. After routine OT profile investigations and pre-anesthetic check-ups, written and informed consent was taken from all the patients.

Surgical technique

The patients underwent surgery in a beach chair position under general anesthesia. Delto's pectoral approach was used for all the patients. Postoperatively shoulder arm pouch was advised for the patients, and six doses of antibiotics were given.

Starting at the coracoid process, an 8 cm to 10 cm incision was performed along the course of the deltopectoral groove. The inter-nervous planes of the deltoid and pectoralis major muscles were identified and separated. Depending on the exposure, the cephalic vein was retracted medially or laterally. External rotation was employed to tense the subscapular muscle, incised along the fibers' course. The fracture's remnants were identified, and the hematoma was removed [6]. Tag sutures were put in the rotator cuff muscles, which would be removed later.

K wires were used to make a preliminary reduction, confirmed in both orthogonal views. The PHILOS plate was placed 5-8 mm posterior to the bicipital groove and 2-4 mm distal to the larger tuberosity. Using the woodpecker technique, the plate was first fastened to the distal fragment, and screws were inserted into the head [7,8].

An immediate check x-ray was obtained to evaluate the alignment of the bones and confirm that the reduction was ideal. On the first post-operative day, passive shoulder mobilization began. On the fourteenth day, the wound was inspected, and the staples were removed. Active shoulder mobilization was started after two weeks. The Constant Murley shoulder score was used to assess functional ability.

Four-week, six-week, three-month, and subsequent follow-ups were conducted. The Murley shoulder score was rated as poor (0-55), moderate (56-70), good (71-85), and excellent (86-100). At each follow-up, radiographs were collected to assess fracture union, and any complications such as fracture displacement, loss of reduction, varus, and valgus angulation were noted. Backing out of the screw, plate breakage/pull-out, malunion, nonunion, or avascular necrosis of the humeral head were all failures.

## Results

The mean age of our forty patients was 46.8 (20-70), with a female: male ratio of 1:2.3. Twenty-seven patients belonging to the more than 45 age group suggested a strong relation of proximal humerus osteoporosis, which is age-related. The majority of patients were injured as a result of a traffic collision (70%), followed by a fall from a height (17.5%) and other reasons (12.5 percent ). Thirty cases involved the dominant side (75%). Eight patients had 1 part fracture (20%), 14 (35%) had 2-part fractures, 14 (35%) had 3-part fractures, and four (10%) had 4-part fractures, according to Neer. The description of the study participants is summarised in Table 1.

**Table 1:** Description of study participants RTA- road traffic accident

Characteristics	Frequency (%)
Age groups	
20 – 45 years	13 (32.5%)
46 – 70 years	27 (67.5%)
Sex	
Male	28 (70.0%)
Female	12 (30.0%)
Diagnosis	
1 Part	8 (20.0%)
2 Part	14 (35.0%)
3 Part	14 (35.0%)
4 Part	4 (10.0%)
Side Affected	
Left	10 (25.0%)
Right	30 (75.0%)
Mode of Injury	
RTA	28 (70.0%)
Fall from height	7 (17.5%)
Assault	3 (7.5%)
Shock	2 (5.0%)
Mean (SD) duration of fracture union	13.75 (2.50)
Median (IQR) fracture union	14 (12 – 16)

Fractures united at an average of 13.75 weeks. The functional outcome was excellent in 2 patients (5%), good in 22 patients (55%), fair in 7 patients (17.5%), and poor in 9 patients (22.5%). The Constant mean score achieved was 68.75±14.03. We found that Neer type I fractures had the highest Constant scores, 71.62±11.68. A comparison of the fracture type and Constant score is summarised in Table 2. However, the p-value was insignificant for the fracture type and the constant score (0.905).

**Table 2:** Comparison of Type of fracture and Constant score

Type of fracture	Mean Constant Score (SD)	Excellent	Good	Fair	Poor
1 Part	71.62 (11.68)	1 (12.5%)	5 (62.5%)	1 (12.5%)	1 (12.5%)
2 Part	69.71 (16.04)	1 (7.4%)	7 (50.0%)	3 (21.4%)	3 (21.4%)
3 Part	65.85 (14.42)	0	7 (50.0%)	3 (21.4%)	4 (28.6%)
4 Part	69.75 (12.65)	0	3 (75.0%)	0	1 (25.0%)
Total	68.75 (14.03)	2 (5.0%)	22 (55.0%)	7 (17.5%)	9 (22.5%)
p-value	0.810	0.905			
Test performed	One-way ANOVA	Chi-square test			

Four patients developed a superficial infection treated with antibiotics, and one had developed stiffness of the shoulder joint. Five patients developed implant failure, including avascular necrosis in 3 patients (7.5%) and screwed back out in 2 patients (5%). The complications are listed in table 3.

**Table 3:** Complications

Complications	Poor	Moderate	Good	Excellent	Total
Superficial infections	1 (11.1%)	1 (14.3%)	2 (9.1%)	0	4 (10.0%)
Avascular necrosis	3 (13.4%)	0	0	0	3 (7.5%)
Screw backout	1 (11.1%)	0	1 (4.5%)	0	2 (5.0%)
Stiffness of shoulder	1 (11.1%)	0	0	0	1 (2.5%)
Nil Complications	6 (66.7%)	6 (85.7%)	16 (72.7%)	2 (100%)	30 (75.0%)

The constant score was compared between different variables like age groups, sex, fracture pattern, and mode of injury but the values were statistically insignificant (Table 4).

**Table 4:** Comparison of Constant score between different factors

Characteristics	Mean Constant Score	p-value
Age groups		
20 – 45 years	64.23 (15.51)	0.160
46 – 70 years	70.92 (13.01)	
Sex		
Male	68.5 (14.34)	0.942
Female	68.85 (14.16)	
Diagnosis		
1 Part	71.62 (11.68)	0.810
2 Part	69.71 (16.04)	
3 Part	65.85 (14.42)	
4 Part	69.75 (12.65)	
Side Affected		
Left	66.9 (13.7)	0.636
Right	69.36 (14.3)	
Mode of Injury		
RTA	63.33 (22.3)	0.797
Fall from height	66.14 (13.71)	
Assault	69.60 (14.07)	
Shock	74.0 (1.41)	

The mean time of fracture union was 13.75 weeks. A comparison of fracture union with Constant score was summarized in Table 5, but the results were statistically insignificant (p-value - 0.263).

**Table 5:** Comparison of Fracture union and Constant score categories Mean (SD): Fracture union in weeks

Fracture union	Mean (SD)	p-value
Poor	13.5 (1.6)	0.263
Moderate	13.0 (3.2)	
Good	13.8 (2.5)	
Excellent	17.0 (1.4)	
Total	13.75 (2.5)	

## Discussion

Most proximal humeral fractures require conservative treatment. Percutaneous pinning, intramedullary fixation, interfragmentary fixation with sutures, and hemiarthroplasties are some of the surgical options for proximal humeral fractures. Adopting less intrusive fracture reduction and fixation methods has become popular in recent years. The less invasive the treatment, the more operating requirements are required, such as 1) strong bone stock, 2) minimum tuberosity comminution, 3) patient willingness to participate in post-operative physiotherapy regimens, and 4) advanced surgical abilities [9,10].

The use of PHILOS plates and screws to fix proximal humeral fractures has been linked to problems such as subacromial impingement, screw pull-out in osteoporotic bone, and avascular necrosis due to severe periosteal stripping. The use of T-buttress plates in fixing proximal humeral fractures has been linked to a significant rate of fixation failure, according to Kristiansen and Christensen [11]. Wijgman et al. [12] found that 87 percent of patients with three- and four-part fractures treated with T-buttress plates had good intermediate and long-term results. The participants in their study had an average age of 48 years.

Fractures of the Proximal humerus have lately been treated with novel implants such as the plan tan humerus fixator plate, Polaris nail, and PHILOS plate. Two cancellous compression screws in the head of the humerus are combined with a plate on the shaft of the humerus to form the humerus fixator plate. According to Sadowski et al. [13], the use of this device in older patients results in a 100% failure rate. Polaris nails have been demonstrated to be effective in both older and younger patients with 2 part fractures.

This study did not assess definitive osteopenia by testing bone mineral density or using a Z score. However, trivial trauma resulting in proximal humerus fractures is more seen in the age group more than 45 years.

In this work, a new surgical strategy for the treatment of displaced proximal humerus fractures was presented. It combines the ideas of traditional plate fixation with the principles of locking screws. For the proximal humerus, the plate is pre-shaped and contoured [14]. The advantages of this implant include the improved purchase in osteopenic bone, no loss of reduction or varus/valgus angulations, and angular and axial stability of the construct provided by the locking screws in the plate. We obtained preliminary results in terms of functional outcomes using locking plates. The procedure's main technically problematic part is acquiring the correct version of the humerus for proper plate positioning. We had some impingement concerns with the early sample of patients due to the relatively newer implant and the novel technique used. Because this plate does not affect the envelope of the soft tissue, it causes less injury to the vascular supply of the fracture, lowering the likelihood of osteonecrosis [15].

Another difficult element is avoiding impingement of the plate's top on the acromion by putting it very proximal to the humerus. A K wire put through an opening on the top of the plate, which should be in line with the tip of the greater tuberosity, can be used to prevent this. It is done when the plate is initially positioned. Incorrect positioning of the diverging screws in the humeral head might also result from placing the plate too high. When using proximal interlocking screws, be careful not to penetrate the head and cause chondrolysis [16].

Limitations

The main limitation of this study is less follow-up period and a lack of a control group. It is a single-center study, and patient characteristics are not evaluated in this study as they might lead to implant failure. The risk factors such as smoking, diabetes mellitus, or inherent bony disorders were not analyzed in this retrospective study. Definitive osteopenia was not assessed in this study by testing bone mineral density. This study deals only with the functional outcome of managing proximal humerus fractures with PHILOS plating. However, further studies are required to compare the outcomes of various modes of management of proximal humerus fractures and assess the outcomes in definitive osteopenic bones.

## Conclusions

One part fracture has a better functional outcome when compared with other types of fractures. Proximal humerus fractures are more common in males and strongly correlate with osteoporotic bones. The locking plate, we feel, provides adequate fracture stability and facilitates early shoulder mobilization without jeopardizing fracture union. The use of the PHILOS plate as a surgical alternative in the management of displaced proximal humeral fractures is something we would suggest.

## References

[1] Neer CS 2nd (1970). Displaced proximal humeral fractures. II. treatment of three-part and four-part displacement. J Bone Joint Surg Am.

[2] Iannotti JP, Ramsey ML, Williams GR Jr, Warner JJ (2004). Nonprosthetic management of proximal humeral fractures. Instr Course Lect.

[3] Kristiansen B, Kofoed H (1987). External fixation of displaced fractures of the proximal humerus. technique and preliminary results. J Bone Joint Surg Br.

[4] Kristiansen B, Christensen SW (1986). Plate fixation of proximal humeral fractures. Acta Orthop Scand.

[5] Thyagarajan DS, Haridas SJ, Jones D, Dent C, Evans R, Williams R (2009). Functional outcome following proximal humeral interlocking system plating for displaced proximal humeral fractures. Int J Shoulder Surg.

[6] Young TB, Wallace WA (1985). Conservative treatment of fractures and fracture-dislocations of the upper end of the humerus. J Bone Joint Surg Br.

[7] Lind T, Krøner K, Jensen J (1989). The epidemiology of fractures of the proximal humerus. Arch Orthop Trauma Surg.

[8] Wijgman AJ, Roolker W, Patt TW, Raaymakers EL, Marti RK (2002). Open reduction and internal fixation of three and four-part fractures of the proximal part of the humerus. J Bone Joint Surg Am.

[9] Rajasekhar C, Ray PS, Bhamra MS (2001). Fixation of proximal humeral fractures with the Polarus nail. J Shoulder Elbow Surg.

[10] Koukakis A, Apostolou CD, Taneja T, Korres DS, Amini A (2006). Fixation of proximal humerus fractures using the PHILOS plate: early experience. Clin Orthop Relat Res.

[11] Hawkins RJ, Bell RH, Gurr K (1986). The three-part fracture of the proximal part of the humerus. operative treatment. J Bone Joint Surg Am.

[12] Hawkins RJ, Kiefer GN (1987). Internal fixation techniques for proximal humeral fractures. Clin Orthop Relat Res.

[13] Sadowski C, Riand N, Stern R, Hoffmeyer P (2003). Fixation of fractures of the proximal humerus with the PlantTan Humerus Fixator Plate: early experience with a new implant. J Shoulder Elbow Surg.

[14] Goldman RT, Koval KJ, Cuomo F, Gallagher MA, Zuckerman JD (1995). Functional outcome after humeral head replacement for acute three and four part proximal humeral fractures. J Shoulder Elbow Surg.

[15] Moeckel BH, Dines DM, Warren RF, Altchek DW (1992). Modular hemiarthroplasty for fractures of the proximal part of the humerus. J Bone Joint Surg Am.

[16] Resch H, Hübner C, Schwaiger R (2001). Minimally invasive reduction and osteosynthesis of articular fractures of the humeral head. Injury.

